# Serological Screening of the *Schistosoma mansoni* Adult Worm Proteome

**DOI:** 10.1371/journal.pntd.0002745

**Published:** 2014-03-20

**Authors:** Fernanda Ludolf, Paola R. Patrocínio, Rodrigo Corrêa-Oliveira, Andréa Gazzinelli, Franco H. Falcone, André Teixeira-Ferreira, Jonas Perales, Guilherme C. Oliveira, Rosiane A. Silva-Pereira

**Affiliations:** 1 Centro de Pesquisas René Rachou-Fiocruz/MG, Genomics and Computational Biology Group, Belo Horizonte, Minas Gerais, Brazil; 2 National Institute of Science and Technology in Tropical Diseases – INCT-DT, Brazil; 3 Centro de Pesquisas René Rachou-Fiocruz/MG, Cellular and Molecular Immunology Laboratory, Belo Horizonte, Minas Gerais, Brazil; 4 Universidade Federal de Minas Gerais, Nursing School, Belo Horizonte, Minas Gerais, Brazil; 5 The University of Nottingham, School of Pharmacy, Division of Molecular and Cellular Science, Nottingham, East Midlands, United Kingdom; 6 Fiocruz/RJ, Department of Physiology and Pharmacodynamics, Toxicology Laboratory, Rio de Janeiro, Rio de Janeiro, Brazil; Swiss Tropical and Public Health Institute, Switzerland

## Abstract

**Background:**

New interventions tools are a priority for schistosomiasis control and elimination, as the disease is still highly prevalent. The identification of proteins associated with active infection and protective immune response may constitute the basis for the development of a successful vaccine and could also indicate new diagnostic candidates. In this context, post-genomic technologies have been progressing, resulting in a more rational discovery of new biomarkers of resistance and antigens for diagnosis.

**Methodology/Principal Findings:**

Two-dimensional electrophoresed *Schistosoma mansoni* adult worm protein extracts were probed with pooled sera of infected and non-infected (naturally resistant) individuals from a *S. mansoni* endemic area. A total of 47 different immunoreactive proteins were identified by mass spectrometry. Although the different pooled sera shared most of the immunoreactive protein spots, nine protein spots reacted exclusively with the serum pool of infected individuals, which correspond to annexin, major egg antigen, troponin T, filamin, disulphide-isomerase ER-60 precursor, actin and reticulocalbin. One protein spot, corresponding to eukaryotic translation elongation factor, reacted exclusively with the pooled sera of non-infected individuals living in the endemic area. Western blotting of two selected recombinant proteins, major egg antigen and hemoglobinase, showed a similar recognition pattern of that of the native protein.

**Concluding/Significance:**

Using a serological proteome analysis, a group of antigens related to the different infection status of the endemic area residents was identified and may be related to susceptibility or resistance to infection.

## Author Summary

Despite intensive efforts towards disease control, schistosomiasis is still highly prevalent in most endemic countries. Although effective treatment is available and widely used, it does not prevent reinfection, as it could be achieved with the use of a vaccine. Efforts to control and eradicate schistosomiasis rely on praziquantel, the only drug available for treatment. Therefore, the identification of antigens that can induce protective immunity is highly desirable, as well as the need for more sensitive assays, useful to detect low intensity infections and treatment follow-up. The occurrence of natural resistance in schistosome endemic areas suggests that there is protective immunity. However, the mechanisms involved in protection, or the proteins that induce this protective immunity, are not yet known. These proteins, once identified, may constitute the basis for a successful vaccine. In this study, we compared the profile of reactive proteins to the serum antibodies of infected and non-infected individuals residing in a schistosomiasis endemic area using two-dimensional western blotting. The association of proteomic and serological screening methodologies enabled the identification of immunogenic proteins of the parasite, which could be an informative source for the development of vaccines and new diagnostic assays. In this manuscript we describe the discovery of potential candidate proteins for subsequent testing as protective or diagnostic antigens.

## Introduction

Schistosomiasis is one of the most important parasitic diseases, being prevalent in 76 countries [1]. Despite many control efforts, mainly after the introduction of a chemotherapeutic treatment in 1980s, the disease is still highly prevalent [2]. The control of the main medically important species *Schistosoma mansoni*, *Schistosoma japonicum and Schistosoma haematobium* is based on the use of praziquantel, the only drug available for chemotherapy [3]. The use of the chemotherapy has a clear effect on morbidity [4], [5]. However, repeated mass drug administration has exerted selective pressure on parasite population and resistance to praziquantel is being described by different investigators [6].

The development of long-term protection based on vaccination would be of significant benefit for disease control [7]. Despite a large body of research in this area and one ongoing clinical trial [8], there is no effective vaccine against schistosomiasis. Together with the fact that mass drug administration has been applied widely and the increasing drug pressure on the parasite population, it becomes more evident the need to find alternative methods of schistosomiasis control/elimination. In this context development of an effective vaccine is a plausible alternative.

The lack of understanding of the protective immunological mechanisms, and the difficulty in identifying antigens which stimulate such a response, remain the major barriers towards the development of anti-schistosome vaccines [9]. Many single antigens with potential use as a vaccine have been proposed, but most have showed disappointing results even with different immunization schemes and experimental models [10], [11]. Nevertheless, distinct observations in animals and humans indicate that it is feasible to achieve protection against infection. Significant levels of protection were obtained in experiments with irradiated cercariae [12] and with some recombinant antigens [13]–[16]. Furthermore, several reports from our group and others have suggested that resistance to infection is acquired naturally or drug induced [17]–[21]. In our studies specifically, we have shown that resistance may develop naturally in endemic areas, describing a group of individuals, that live in areas where transmission is active but do not get infected, called Endemic Normals [22]. These individuals were defined using specific criteria such as being *S. mansoni* egg-negative over 5 years despite continuous exposure to contaminated water, no previous treatment with anthelmintic drugs and having vigorous cellular and humoral immune response to crude schistosome antigen preparations [23], [24]. The immune response of individuals with natural resistance to schistosomiasis differs significantly from that of post-treatment resistant and infected individuals [17].

The immunological mechanisms that prevent the infection in drug-induced and naturally resistant individuals living in endemic areas for schistosomiasis may constitute the basis for the development of a successful vaccine [7], [25], [26]. Therefore, we believe that using the most recent technology to identify antigens reactive to antibodies from resistant individuals, both natural and drug induced, we will be able to screen at a much faster pace for putative protective antigens.

Schistosomiasis control will benefit from a vaccine, but a new generation of diagnostic tools is as much a part of any control and eradication strategy. Available tools, especially fecal exams, encounter limitations in low parasitic load and low infection rates settings and in the follow-up to treatment [27], [28]. The next generation of assays needs to be simple, inexpensive, fast, sensitive, specific and capable of distinguishing active from prior infection [28], [29].

The empirical science used in the last decades is strikingly changing with the use of high throughput global approaches to a less biased, and more encompassing development for the proposition of new biomarkers to the discovery of vaccine candidates, drug and diagnostic targets [24], [25], [30]. Significant progress has been made in *S. mansoni* and *S. japonicum* proteomic studies, mainly with the description of proteins differentially expressed in the different life cycle stages of the parasite [31]–[33], between male and female worms [34] and irradiated and normal cercariae [35]. Furthermore, proteomic studies have concentrated mainly on the studies of proteins exposed on the parasite surface and readily accessible to the host, i.e. identification of tegumental proteins [36]–[42] or secreted/excreted proteins [38], [43]–[49].

A combination of proteomic and serological analyses has been used as a promising experimental approach for screening new biomarkers candidates to different diseases, identifying proteins useful for diagnosis, therapy and vaccine design [50]–[54]. A limited number of studies have performed serological-proteomic analysis using schistosome proteins. Serum of experimentally infected animals was used to screen antigens of *S. japonicum* in two-dimensional electrophoresis (2-DE) in an attempt to identify suitable antigens for diagnostic purposes, identifying four proteins out of 30 immunoreactive protein spots [30]. Comparative proteomic and immunological analysis of *S. haematobium*, *S. bovis* and *Echinostoma caproni* revealed some common cross-species antigens and species-specific targets [55]. Additional studies using *S. mansoni* immunoprecipitated proteins from protective and non-protective rat serum analyzed by 2-DE showed four spots specifically reactive with the protective rat serum [56]. High and low worm burden serum from infected Rhesus macaques were used to probe *S. mansoni* gut secretions and tegument surface proteins in 2-DE. The study identified gut digestive enzymes, tegument surface hydrolases and antioxidant enzymes as IgG targets of the IgG high titer serum of low burden animals [57]. The use of infected human serum was conducted only for *S. haematobium*, where a total of 71 immunoreactive protein spots were identified as 26 different proteins [58]–[60].

Although important observations have been made in relation to schistosome antigen identification, a human schistosomiasis mansoni coherent screening for new antigens is still necessary. In the present study we used, for the first time, serum antibodies of infected and naturally resistant individuals from a *S. mansoni* endemic area to compare the recognition profiles of adult worm antigens by these serum antibodies using two-dimensional Western blotting. We identified a total of 47 *S. mansoni* antigenic proteins. We also observed that some of the antigens were differentially recognized by antibodies of infected and naturally resistant individuals. This panel of antigens may constitute an informative source for the improvement of diagnostic tools and vaccine development to schistosomiasis.

## Materials and Methods

### Ethics statement

This research was approved by the Ethics Committee for Human Research of CPqRR – FIOCRUZ (CAAE: 1.0.245.000-08). Written informed consent was obtained from all participants at the time of the stool collection.

All procedures involving animals were conducted in compliance with the Manual for the Use of Animals/FIOCRUZ and approved by the Ethics Committee on the Use of Experimental Animal (CEUA – FIOCRUZ) license number LW-17/09.

### Biological material

BALB/c mice were infected by the subcutaneous route with 100 *S. mansoni* cercariae of the LE strain. After 45 days, adult worms were recovered by perfusion of the portal mesenteric system, as described by Pellegrino and Siqueira [61]. The adult worms were washed three times in RPMI medium (SIGMA), snap frozen in liquid nitrogen and stored at −70°C until use.

### Preparation of protein extracts

*S. mansoni* adult worm total protein extract (AW-TOT) was obtained from direct lysis of the parasites in lysis buffer [8 M Urea, 2 M Thiourea, 4% 3-3-Cholamidopropyl-dimethylammonio-propane-sulfonate (CHAPS), 50 mM dithiothreitol (DTT), 20 mM Tris and Complete Mini Protease Inhibitor Cocktail Tablets (Roche)]. After homogenization under continuous agitation for 2 hours at room temperature, followed by 10 repeated passages through a 30-gauge hypodermic needle, the homogenate was centrifuged at 20,000×g for 30 min at 25°C and the supernatant was collected and stored at −70°C until use.

*S. mansoni* adult worm tegument protein extract (AW-TEG) was obtained by freeze/thaw/vortex method in Tris Buffered Saline (TBS) supplemented with Complete Mini Protease Inhibitor Cocktail Tablets, according to Roberts and co-workers [62] with some modifications. Briefly, after thawing on ice, the outer tegumental membrane complex was removed by ten 1 second vortex pulses at maximum speed. All the supernatant content, obtained after decanting the stripped worms, was passed through a 30-gauge hypodermic needle 10 times and then concentrated using a 3 kDa cutoff centrifuge filter (Millipore). Next, acetone precipitation was performed and the pellet was solubilized in SBI buffer [7 M Urea, 2 M Thiourea, 15 mM 1,2-diheptanoyl-*sn*-glycero-3-phosphatidylcholine (DHPC), 0.5% Triton X-100, 20 mM DTT and Complete Mini Protease Inhibitor Cocktail Tablets], as described by Babu and co-workers [63], and stored at −70°C until use.

Protein concentration of both protein extracts was measured by the Bradford method [64] and the quality of the extracts was verified by SDS-PAGE 12% [65].

### Human serum samples collection

The human serum samples were obtained from a rural population of the Virgem das Graças village (VDG). This is a hyperendemic area for schistosomiasis located in the Jequitinhonha Valley in northern Minas Gerais State, Brazil. In VDG there is no treated water or basic sanitation and water contact was determined by direct observation and translated to Total Body Minutes (TBM). Stool samples were processed by the Kato-Katz method to detect eggs of *S. mansoni* and other intestinal helminthes. Two slides from each stool sample collected in three consecutive days were used for quantification of number of eggs/gram of stool [66].

In the present study, the serum samples were chosen according to the following criteria: individuals not infected by other helminthes (*Ascaris lumbricoides*, *Trichuris trichiura* and *Ancylostoma*), between 20–50 years of age, man or non-pregnant women. Individuals positive for *S. mansoni* eggs at the start of the study in 2001 were called Infected Individuals (INF), and those who were egg negative in the three years of the study (2001, 2002 and 2006) were identified as Non-Infected Individuals from Endemic Area (NE). All the serum samples used in this study were obtained at the initiation of the study in January 2001, before mass chemotherapy with praziquantel was administered. Serum samples of 13 INF, who had 8 to 304 eggs/gram of stool, and 9 NE were used (Table 1). Sera of Non-Infected volunteers from non-endemic sites (NI) were also used in this study, 7 from USA or 2 from UK sites.

**Table 1:** Selected volunteers from the endemic area of schistosomiasis, Virgem das Graças, Brazil.

Volunteers ID	Localization	Gender	Age	EPG	TBM
***Infected*** ** (n = 13)**					
31	cVDG	F	25	88	229
58	cVDG	F	33	8	157
128	cVDG	M	28	200	91
129	cVDG	F	33	304	126
189	cVDG	F	31	48	117
214	cVDG	F	35	80	187
238	cVDG	F	34	20	284
329	Suss	F	38	8	126
356	Card1	M	37	48	184
371	Card1	F	26	160	163
403	Card2	M	36	152	118
493	Card3	F	49	43	143
514	Card3	F	43	212	89
***Non-Infected*** ** (n = 09)**					
62	cVDG/Card2	M	33	0	155
83	cVDG	M	27	0	107
115	cVDG	M	46	0	93
233	cVDG	F	40	0	156
294	Suss	F	24	0	-
312	Suss	M	37	0	88
366	Card1	M	37	0	-
445	Card2	F	49	0	124
609	cVDG	F	28	0	53

Volunteers ID: identity number of the selected volunteers. Localization: sites of volunteers in the village (cVDG: central village of Virgem das Graças; Suss: Suçuarana; Card1/2/3: Cardoso 1, 2 or 3). Gender: M-Male and F-Female. Age: age of volunteers at the serum collection in 2001. EPG: Eggs Per Gram of faeces, from 3 samples, 2 slides each, by Kato-Katz method. TBM: Total Body Minutes, time in minutes of body exposure to water. (-): missing data.

### Two-dimensional electrophoresis (2-DE)

For each two-dimensional-polyacrylamide-gel-electrophoresis (2D-PAGE), 100 µg of proteins from AW-TOT or AW-TEG extracts were used. The AW-TOT proteins were solubilized in IEF rehydration buffer [8 M Urea, 2 M Thiourea, 4% CHAPS, 0.0025% bromophenol blue, 65 mM DTT and 1% BioLyte 3–10 buffer 100× (Bio-Rad)] and the AW-TEG proteins in SBI buffer supplemented with 0.0025% bromophenol blue and 0.4% BioLyte 3–10 buffer 100× (Bio-Rad) [63], both to 125 µl final volume. After homogenization under continuous agitation for 1 hour at room temperature, the samples were centrifuged at 16,000×g for 30 min. The supernatants were loaded onto 7 cm IPG strip 3–10, 3–10NL or 5–8 pH ranges (Bio-Rad) by in-gel sample rehydration. Isoelectric focusing was carried out in a Protean IEF Cell (Bio-Rad) at 20°C and 50 µA/strip. Passive rehydration was performed for 4 hours, followed by active rehydration at 50 V for 12 hours, and focalization at 500 V for 30 min, followed by 1,000 V for 30 min, 4,000 V for 1 hour and 4,000 V up to 16,000 V/h. The IPG strips were equilibrated in reducing buffer (6 M Urea, 30% glycerol, 2% SDS, 50 mM Tris-HCl pH 8.8, 0.001% bromophenol blue and 130 mM DTT) for 10 min, and in alkylating buffer containing 135 mM iodoacetamide for a further 10 min. The IPG strips and molecular weight standard were placed on top of 12% SDS-PAGE gels and sealed with 0.5% agarose. The second dimension electrophoretic protein separation was carried out using a Mini-Protean III (Bio-Rad) under 60 V constant voltage for 10 min, and then under 100 V until the dye front reached the bottom of the gel.

For each 2-DE experiment, at least two 2D-PAGEs were performed in parallel, one to be used in a Western blotting experiment and another corresponding 2D-PAGE to be stained by Colloidal Coomassie Blue G-250 for spot excision and protein identification.

### Two-dimensional western blotting (2D-WB)

The 2D-PAGEs were immediately transferred to a PVDF-based membrane (Immuno-blot 0.2 µm, Bio-Rad) using a Trans-Blot Electrophoretic Transfer Cell (Bio-Rad) at 100 V (2–3 mA cm^2^) for 120 min with transfer buffer (25 mM Tris-Base, 192 mM glycine, 20% methanol). The membranes were washed in water and air-dried. Before proceeding with Western blotting, the membranes were re-activated in 100% methanol and blocked for 16 hours in TBS (20 mM Tris-HCl, 500 mM NaCl, pH 7.5) containing 0.05% Tween-20 and 3% BSA (TBS-T/3% BSA) at room temperature. Each membrane was incubated separately for 2 hours with each pool of INF, NE or NI (from USA volunteers) sera diluted 1∶500 in TBS-T/1% BSA. After 2×30 min washes in TBS-T/1% BSA, the membranes were incubated with goat anti-human Ig's polyvalent antibody, HRPO conjugated (Caltag Laboratories), diluted 1∶100,000 in TBS-T/1% BSA. After 2×30 min washes in TBS-T and 1×15 min wash in TBS, the immunoreactive proteins were developed using ECL Plus Western Blotting Detection System (GE Healthcare) and the membranes were exposed for 16 hours to X-Ray film. All the 2D-WB experiments were performed in triplicate.

### In-gel digestion

The X-Ray films and its corresponding Colloidal Coomassie Blue stained 2D-PAGE were overlapped. The antigenic protein spots were manually and individually excised from the corresponding 2D-PAGE for mass spectrometry identification. First, spots were washed in Milli-Q water, and then destained 2×15 min in 50% acetronitrile (ACN)/25 mM ammonium bicarbonate (AB) pH 8.0 until clear of blue stain. The gel fragments were dried in 100% ACN for 5 min, followed by rehydration in 100 mM AB for 5 min and addition of same volume of 100% ACN. The solution was removed and 100% ACN was added again. After removing the ACN, the spots were completely dried in a Speed Vac Concentrator Plus (Eppendorf) for 20 min. The final dried spots were re-swollen in 10 µl of 20 µg/ml Sequencing Grade Modified Trypsin (Promega) in 25 mM AB for 10 min and then, additional 10 µl of 25 mM AB were added. Protein digestion was conducted at 37°C for 16 hours. After the incubation, the supernatant was transferred to a clean tube and 30 µl of 5% formic acid (FA)/60% ACN were added to gel spots for the extraction of the tryptic peptides. This procedure was performed 2×30 min under constant agitation. The supernatant was pooled to the respective tube containing the initial peptide solution. This solution was dried in a Speed Vac and the peptides were resuspended in 8 µl of 0.1% FA. The peptides were desalted in reverse phase micro-columns Zip Tip C18 (Millipore), according to manufacture instructions. Peptides were dried again and resuspended in 1 µl of 50% ACN/0.1% trifluoracetic acid (TFA) solution.

### Protein identification by mass spectrometry

MALDI-ToF-ToF analysis was performed on the 4700 Proteomics Analyzer (Applied Biosystems). Briefly, 0.5 µl of the micro-column eluate was mixed with 0.2 µl of alpha-cyano-4-hydroxycinnamic acid matrix (20 mg/ml in 30% ACN/0.3% TFA). Samples were spotted onto the ABI 192-targed MALDI plate by co-crystallization and mass spectrometry data were acquired in positive and reflectron mode, mass range 900–4,000 Da, using a neodymium-doped yttrium aluminum garnet (Nd: YAG) laser with a 200-Hz repetition rate. Typically, the analyses were conducted using 2,000 shots of MS and 4,000 shots of MS/MS to the 10 most abundant ions. External calibration was performed using a mixture of four peptides: des-Arg1-bradykinin (m/z = 904.47), angiotensin I (m/z = 1296.69), Glu1-fibrinopeptide B (m/z = 1570.68) and adrenocorticotropic hormone (18–39) (m/z = 2465.20) (mass standards kit for the 4700 Proteomics Analyzer). The list of peptide and fragment mass values generated by the mass spectrometer for each spot were submitted to a MS/MS ion search using MASCOT (Matrix Science, Boston, MA) to search in the NCBInr database. The parameters used were: allowance of two tryptic miss cleavages, peptide error tolerance of ±0.6 Da, MS/MS error tolerance of ±0.2 Da, peptide charge +1 and variable modifications of methionine (oxidation), cysteine (carbamidomethylation and propionamidation). To avoid random matches, only ions with individual score above of the indicated by the MASCOT to identity or extensive homology (p<0.05) were considered for protein identification. To those protein matches obtained from NCBInr search that did not retrieve a Smp number, a blastp search was conducted using the SchistoDB database Version 2.0 (www.schistodb.net) [67].

### Clustering analysis of immunoreactive proteins

Hierarchical clustering was performed with the identified immunoreactive proteins according to similarities in recognition profile with each pool of serum used in this study, in three two-dimensional Western blotting assays. Recognition profile similarity was measured by the Euclidean distance, with complete-linkage among samples, using the Hierarchical Clustering (HC) algorithm available at the GenePattern Platform [68].

### Cloning, *in vitro* expression and western blotting analysis of the recombinant *S. mansoni* hemoglobinase and major egg antigen

The *S. mansoni* hemoglobinase precursor (Smp_075800) and major egg antigen (Smp_049300.3) were expressed using a wheat germ cell-free expression system (TNT SP6 High-Yield Wheat Germ Protein Expression System, Promega) by coupled *in vitro* transcription-translation. The coding region of the corresponding genes were obtained by PCR amplification using a previously constructed *S. mansoni* adult worm cDNA library as template. Primers were designed using the Flexi Vector Primer Designer Tool (Promega), adding an extra C-terminal histidine tag. The primers used to amplify the coding region of *major egg antigen* and *hemoglobinase* genes were as follow: forward (5′-GGCTGCGATCGCCATGTCTGGTGGGAAACAACATA-3′) and reverse (5′-TGATGTTTAAACGTGGTGGTGGTGGTGGTGAGTAATTGCATGTTGCTT-3′), and, forward (5′-CCTGGCGATCGCCATGGTATCCGATGAAACTGTTAGTGA-3′) and reverse (5′- TGATGTTTAAACGTGGTGGTGGTGGTGGTGACCGCAAATTTTTATGATTGCT-3′), respectively. PCR reactions were composed of 25 µl JumpStart REDTaq ReadyMix Reaction Mix (Sigma-Aldrich), 2.5 µl of each gene specific forward and reverse primer (10 µM), 2 µl of *S. mansoni* adult worm cDNA library in 50 µl final volume. The purified PCR products were inserted into pF3A WG (BYDV) Flexi Vector (Promega) using the Flexi Vector System (Promega) and the plasmids were transformed into electrocompetent DH5α *Escherichia coli* cells by electroporation. Colonies were selected and grown in LB-Amp broth. Plasmids were purified using QIAprep Spin Miniprep Kit (Qiagen) and verified by DNA sequencing using the SP6 and T7 terminator primers (Source Bioscience, Nottingham, UK). Protein synthesis was initiated by adding the DNA plasmids as template according to the instructions described in the TNT SP6 High-Yield Wheat Germ Protein Expression System protocol. Protein expression was analyzed by the incorporation of labeled lysine residues (FluoroTect Green_Lys_, Promega) loading 5 µl of the reaction in 4–20% Mini-PROTEAN TGX Precast Gel (Bio-Rad) and detecting with a laser-based fluorescent gel scanner (Fujifilm LAS-4000 Imaging System).

For Western blotting analyzes, 3 µl of the protein synthesis reaction were blotted onto 0.45 µm nitrocellulose membranes after electrophoresis. The membranes were blocked for 16 hours in TBS-T/3% nonfat dry milk and then incubated separately for 2 hours with each pool of INF, NE or NI (from UK volunteers) sera diluted 1∶500 in a pre-adsorbed solution. These serum pools were pre-adsorbed for 16 hours in TBS-T/3% nonfat dry milk and 5% wheat germ protein extract. After 2×30 min washes in TBS-T/1% nonfat dry milk, membranes were incubated with rabbit anti-human IgG antibody (Sigma) diluted 1∶10,000 in TBS-T/1% nonfat dry milk for 1 hour and with anti-rabbit IgG HRP conjugated (ECL Plus Western Blotting Reagent Pack, GE Healthcare) in the same conditions, with 2×30 min washes between antibodies incubations. The membranes were revealed using ECL Plus-Western Blotting Detection System (GE Healthcare) and the proteins were visualized by chemiluminescence detection using a Fujifilm LAS-4000 Imaging System.

## Results

### *Schistosoma mansoni* adult worm antigens recognized by antibodies in pooled sera of infected individuals

*S. mansoni* adult worm total and tegumental protein extracts were used initially in 2D-WB in order to evaluate the antigenicity of proteins using total immunoglobulins in pooled sera of *S. mansoni* infected individuals.

**Figure 1:**
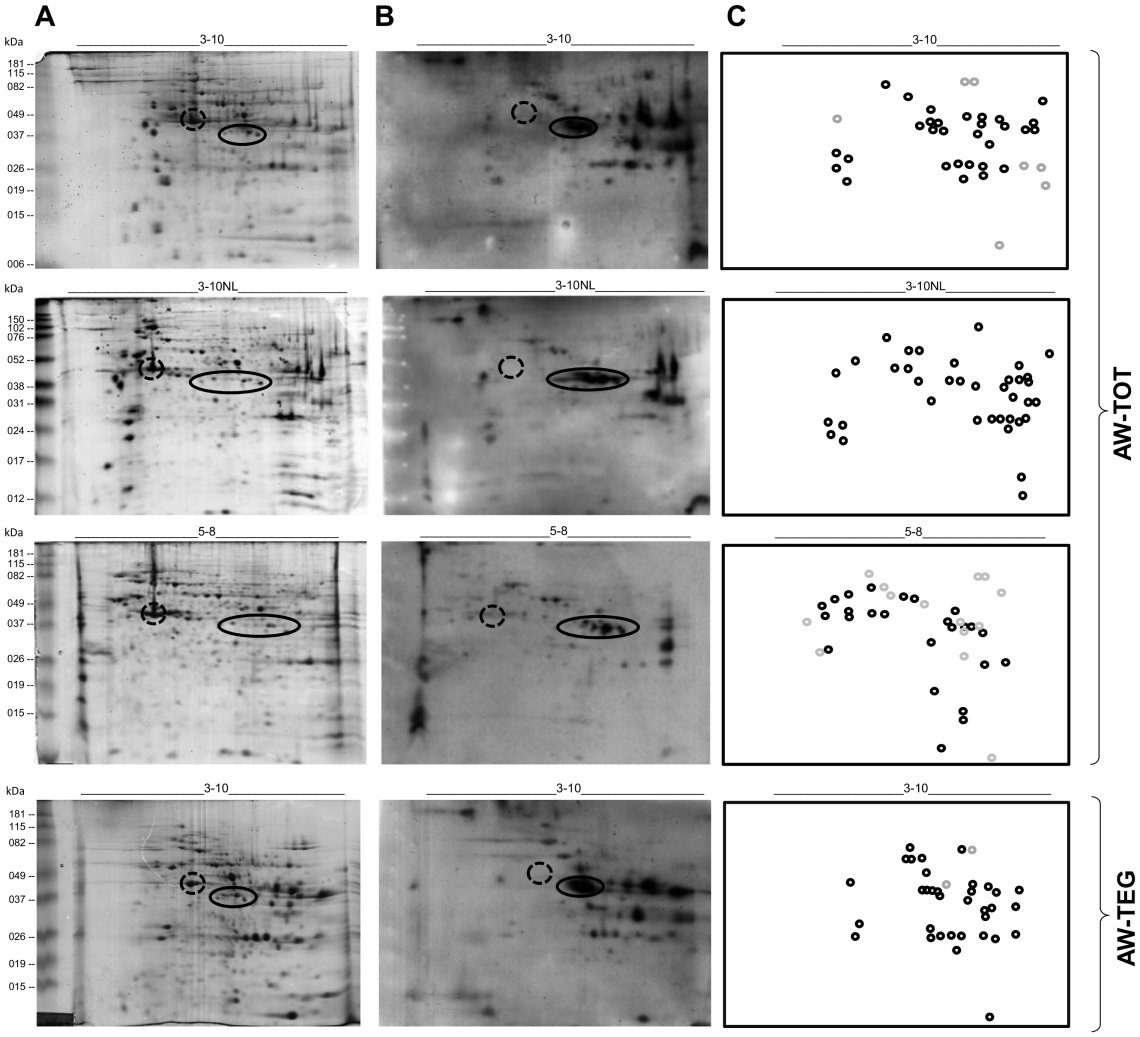
2D-PAGE and 2D-WB of *Schistosoma mansoni* adult worm protein extracts using different IPG strip pH ranges. A) 2D-PAGE of adult worm total (AW-TOT) and tegumental (AW-TEG) protein extracts using 7 cm, pH 3–10, 3–10NL and 5–8 IPG strips and SDS-PAGE 12%, stained by Colloidal Coomassie Blue G-250. B) Corresponding 2D-WB using pool of *S. mansoni* infected individuals serum and anti-human Ig's polyvalent antibody HRPO conjugated. The dashed circle indicates a region of strongly stained protein spots in 2D-PAGE that are weakly or not immunoreactive in 2D-WB and the circle indicates a region of protein spots barely visible in 2D-PAGE and highly immunoreactive. C) Schematic representation of immunoreactive spots excised from the corresponding 2D-PAGE to MS/MS identification. Gray circles represent immunogenic spots not identified and black circles represent immunogenic spots identified by MS/MS. All the identified proteins are listed in Table 2. The figure shows one representative experiment of three replicates.

Firstly, Colloidal Coomassie Blue stained 2D-PAGEs were conducted using different IPG strip pH ranges (3–10, 3–10NL and 5–8) in order to visualize the separation pattern of the AW-TOT protein extract. All the different IPG strip pH ranges used showed good resolution of the spots and minimal streaking. Protein spots were reproducibly resolved in a broad pH range and molecular weight (Figure 1A).

Corresponding 2D-WB using each of the IPG strip pH ranges were performed to determine which pH range would better separate the immunoreactive protein spots when using pool of INF serum against AW-TOT protein extract. The use of IPG strip 3–10, 3–10NL and 5–8 pH ranges showed that distinct antigenic spots were evidenced when resolved by different IPG strip pH ranges (Figure 1B). Although most of the antigenic proteins were common among all the IPG strip pH range used, some proteins were exclusively identified in specific IPG strip pH range, contributing to increase the total number of identified immunoreactive proteins (Table 2).

**Table 2:** Immunoreactive proteins to the pooled sera of individuals from the schistosomiasis endemic area.

PROTEIN DESCRIPTION	GENE ID	IPG strip pH range			
		3–10 3–10 NL	5–8 3–10
		AW-TOT			AW-TEG
14.3.3 epsilon isoform	Smp_034840.3	x	x	x	x
14.3.3 zeta isoform	Smp_009760	x	x		x
Actin-1^1^	Smp_046600	x	x	x	x
Aldehyde dehydrogenase	Smp_050390	x	x	x	x
Antigen Sm 21.7	Smp_086480		x		x
Annexin^2^	Smp_162170.2				x
Arginase	Smp_059980		x		
ATP synthase alpha subunit mitochondrial	Smp_002880.2	x	x		
ATP synthase beta subunit	Smp_038100		x	x	
ATP: guanidino kinase (Smc 74)	Smp_194770		x		
Cathepsin B1 isotype 1	Smp_103610		x		
Cathepsin B1 isotype 2	Smp_067060		x		
Cofilin actophorin	Smp_120700.1			x	
Dynein light chain	Smp_095520			x	
Enolase (Phosphopyruvate hydratase)	Smp_024110	x	x	x	x
Eukaryotic translation elongation factor	Smp_143140				x
Fatty acid-binding protein (Sm14)	Smp_095360.3				x
Filamin	Smp_000100				x
Four and A half lim domains^4^	Smp_048560		x		x
Fructose 1,6-biphosphate aldolase	Smp_042160.2	x	x		x
Gelsolin	Smp_008660.2			x	
Glutathione S-transferase 28 kDa (GST-28)	Smp_054160	x	x	x	x
Glutathione S-transferase 26 kDa (GST-26)	Smp_102070	x	x		
Glyceraldehyde-3-phosphate dehydrogenase	Smp_056970.1	x	x	x	x
Gynecophoral canal protein	Smp_177050		x		
Heat shock protein 70 (HSP70)	Smp_106930	x	x	x	
Hemoglobinase precursor (Antigen Sm32)*	Smp_075800	x	x		x
Hydroxyacylglutathione hydrolase (Glx II)	Smp_091010				x
Major egg antigen*	Smp_049300.3	x	x	x	x
Malate dehydrogenase^3^	Smp_035270.2	x			x
Malate dehydrogenase^3^	Smp_047370		x		
Myosin regulatory light chain	Smp_132670	x	x	x	
Ng,ng- dimethylarginine dimethylaminohydrolase	Smp_052560		x	x	
Phosphoglycerate kinase^5^	gi|1172460	x	x		x
Phosphoglycerate mutase	Smp_096760	x	x	x	x
Protein disulfide-isomerase ER-60 precursor	Smp_079770				x
Reticulocalbin^1^	Smp_147680	x	x		x
*Schistosoma mansoni*, expressed protein	Smp_171780	x	x		
Short-chain dehydrogenase^4^	Smp_081430				x
Superoxide dismutase [Cu-Zn]	Smp_176200.2			x	
Superoxide dismutase [Mn]	Smp_056440				x
Transketolase	Smp_059790.2				x
Trimeric G-protein alpha o subunit	Smp_016630.2			x	
Triose phosphate isomerase	Smp_003990	x	x		
Troponin I (*S. japonicum*)^6^	gil226481381	x			
Troponin T	Smp_179810	x			
Tubulin alpha chain	Smp_016780			x	
**Total number of identified proteins:**	**47**	**22**	**29**	**18**	**25**

All *Smp* IDs can be found in schistodb.net. AW-TOT: adult worm total protein extract, AW-TEG: adult worm tegumental protein extract.

1 : actin was co-extracted from the same spot with reticulocabin, except in pH 5–8 IPG strip;

2 : annexin was co-extracted from the same spot with one of major egg antigen;

3 : the same protein corresponding to different spots and protein sequences;

4 : short-chain dehydrogenase was co-extracted from the same spot with four and A half lim domains;

5 : gene ID corresponding to Smp_018890 and Smp_187370;

6 : troponin I from *S. japonicum*;

*: proteins selected to *in vitro* recombinant protein expression.

AW-TEG protein extract was also used in an attempt to enrich the analysis with immunologically exposed parasite proteins. The AW-TEG protein extract was separated in pH 3–10 IPG strip and it was observed a distinct 2D-PAGE and 2D-WB pattern to the AW-TOT protein extract, although there are some common immunoreactive protein spots in both extracts (Figure 1).

When the 2D-WB X-Ray films and the corresponding Colloidal Coomassie Blue stained 2D-PAGEs were overlapped it was observed that there was no direct correlation between the amount of protein in the AW-TOT and AW-TEG protein extracts and its antigenicity level. Although the most of immunoreactive spots recognized by the INF serum were visible in its corresponding 2D-PAGE, there were some strongly stained protein spots that showed weak or no immunoreactivity (dashed circles in Figures 1A and 1B). Conversely, there were some barely visible protein spots in the 2D-PAGE that were highly immunoreactive (circles in Figures 1A and 1B). Most of the immunoreactive protein spots show a pI above 6.5 and molecular weight above 25 kDa.

Immunoreactive spots visualized in each of the pH ranges were excised from the corresponding Colloidal Coomassie stained 2D-PAGE for proteins identification by mass spectrometry (MS/MS). A total of 37 immunoreactive spots were excised from the 2D-PAGE using AW-TOT protein extract and pH 3–10 IPG strip. Additional 37 and 39 spots were excised from the 2D-PAGE using the pH 3–10NL IPG strip and the pH 5–8 IPG strip, respectively. From the 2D-PAGE using AW-TEG protein extract, 36 immunoreactive spots were excised. Some of the protein spots could not be identified by MS/MS. Using AW-TOT protein extract and pH 3–10 and pH 5–8 IPG strips, 7 and 14 immunoreactive spots were not MS/MS identified, respectively. Two immunoreactive spots from the 2D-PAGE using AW-TEG protein extract were also not identified (gray circles in Figure 1C).

**Figure 2:**
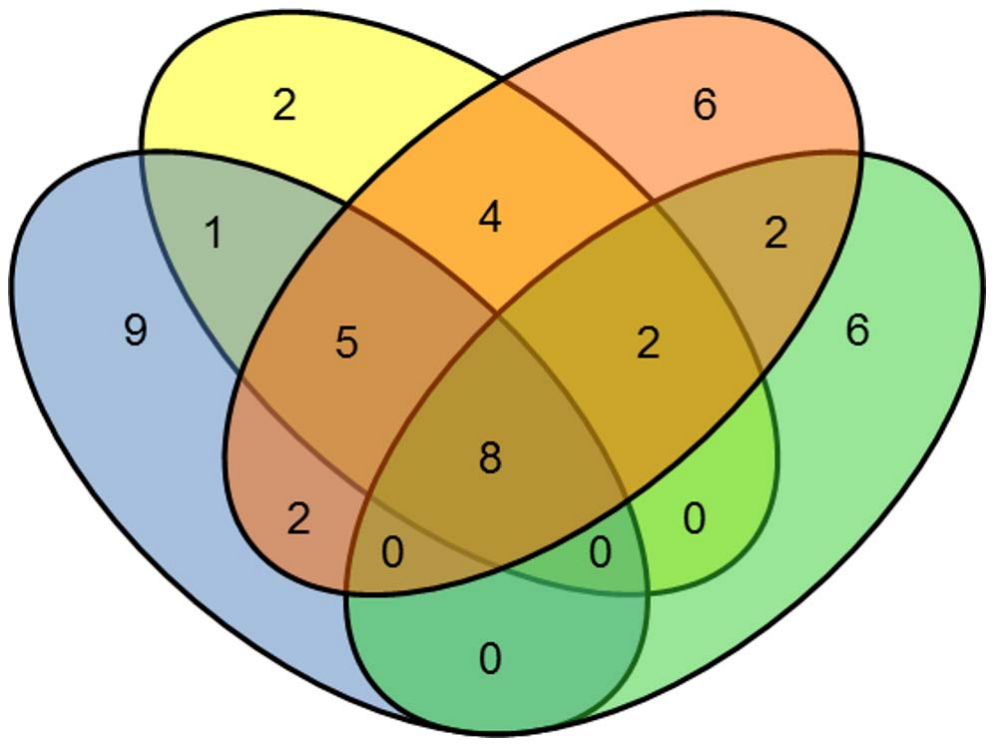
Venn diagram showing the number of unique and shared proteins identified between and among 2D-WB assays. The number of immunoreactive proteins identified using AW-TOT protein extract and pH 3–10 IPG strip are represented in the yellow circle, using pH 3–10NL IPG strip in red and using pH 5–8 IPG strip in green. Spots using AW-TEG protein extract and pH 3–10 IPG strip are represented in the blue circle. Common spots identified between and among the assays are represented overlapped by the circles.

A total of 47 different *S. mansoni* immunoreactive proteins were identified. Using AW-TOT protein extract 22, 29 and 18 proteins were identified from 2D-PAGE of pH 3–10, pH 3–10NL and pH 5–8, respectively. AW-TEG protein extract yielded 25 proteins identified from 2D-PAGE of pH 3–10. Most proteins were identified in more than one pH range, but others were exclusive. Additionally, 9 immunoreactive proteins were identified only in AW-TEG protein extract (Figure 2). All proteins identified by mass spectrometry in at least one 2D-WB experiment were included (Table 2). In some cases, as the result of post-translational modifications, splice variants or paralogue genes, for example, the same protein description was identified for different immunoreactive spots, but a representative gene ID was used.

### Comparative serology of *Schistosoma mansoni* adult worm proteins

In order to identify antigens differentially recognized by antibodies from pooled sera of *S. mansoni* infected (INF) and non-infected (NE) individuals from the endemic area, 2D-WB experiments were performed using both serum pools. A serum pool of non-infected individuals from a non-endemic area (NI) was also used. AW-TOT and AW-TEG protein extracts were focused using pH 3–10 IPG strips. Four 2D-PAGEs were electrophoresed simultaneously. Three of them were used in the 2D-WB with the three serum pools separately (INF, NE, NI). The fourth gel was stained with Colloidal Coomassie for spot excision and MS/MS identification.

**Figure 3:**
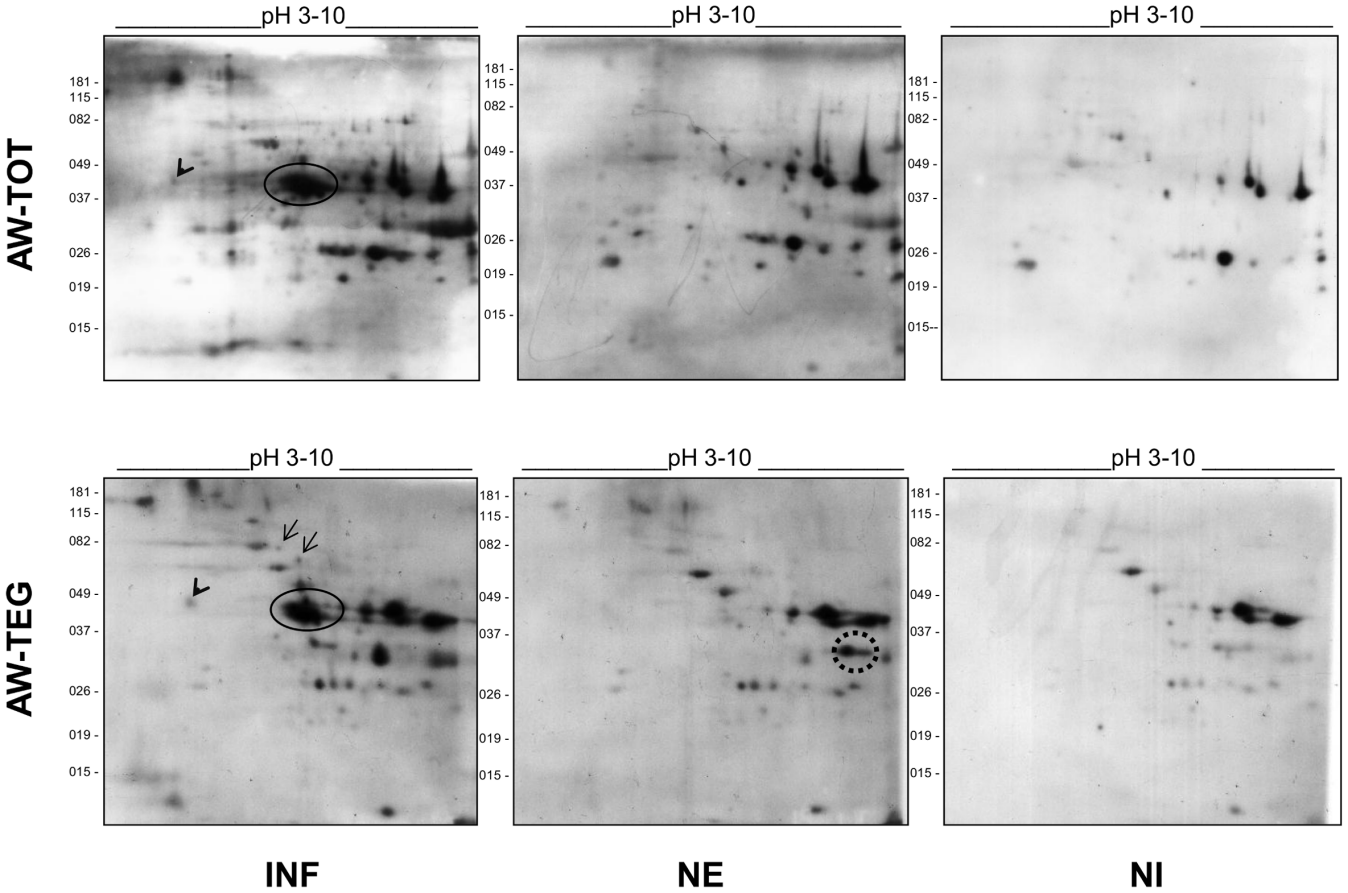
2D-WB of *Schistosoma mansoni* adult worm total and tegumental protein extracts using pooled sera of infected, non-infected from endemic area and non-infected from non-endemic area individuals. Adult worm total (AW-TOT) and tegumental (AW-TEG) protein extracts were separated by 2-DE using 7 cm pH 3–10 IPG strips and SDS-PAGE 12%. The proteins were blotted onto PVDF membranes and probed with *S. mansoni* infected (INF), non-infected from endemic area (NE) and non-infected from non-endemic area (NI) serum pools, following an additional incubation with anti-human Ig's polyvalent antibody HRPO conjugated. Circle, arrows and arrowhead indicate immunogenic protein spots which reacted exclusively with INF serum pool, while dotted circle indicates immunogenic spot which reacted exclusively with NE pooled sera. The figure shows one representative experiment of three replicates.

Quantitative variations on the reaction intensity of the immunoreactive spots were observed among the 2D-WB using the same dilution of the three pooled sera. However, a similar overall pattern of reactive spots was observed, but with higher signal intensity when using the INF serum pool when compared to the NE, which in turn showed higher signal intensity than NI serum pool (Figure 3).

**Figure 4:**
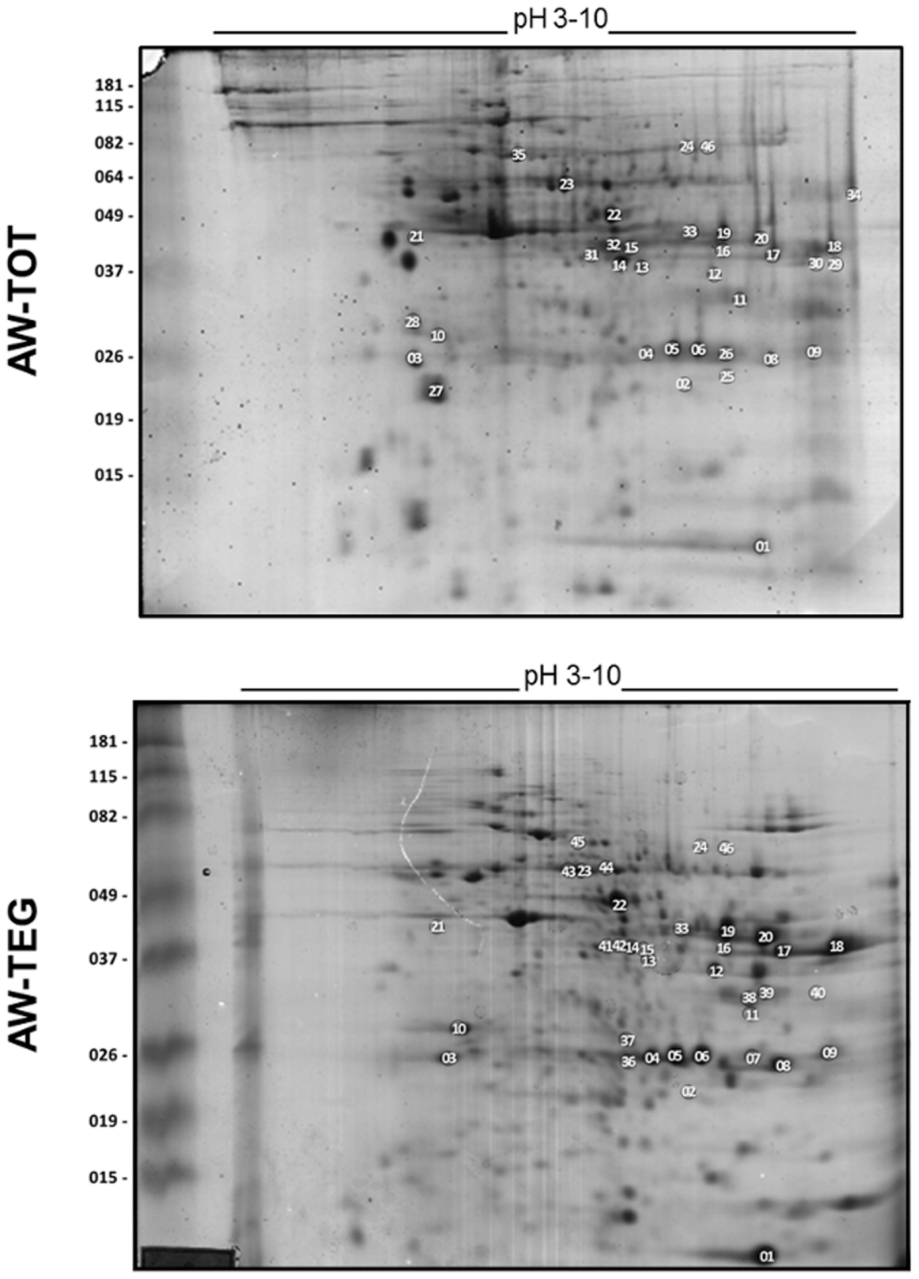
Indication on the 2D-PAGE of the immunoreactive spots identified in 2D-WB experiments. Adult worm total (AW-TOT) and tegumental (AW-TEG) protein extracts were separated by 2-DE using 7 cm pH 3–10 IPG strips followed by SDS-PAGE 12%, and stained by Colloidal Coomassie Blue G-250. Protein spots immunoreactive to INF, NE and/or NI pooled sera on the 2D-WB were extracted from the corresponding 2D-PAGE to mass spectrometry identification. The identified protein spots are numbered according to the Table 3. The figure shows one representative experiment of three replicates.

Qualitative variations of the immunoreactive spots were also visualized among the 2D-WB. Using AW-TOT and AW-TEG protein extracts it was observed that 9 spots were detectable exclusively with the INF serum pool and a single spot exclusively with the NE serum pool (Table 3). From those that reacted only with the INF serum pool, some strong immunoreactive spots of 40 kDa with approximately pI 7.0 were observed, as indicated by a circle in Figure 3. Interestingly, the corresponding spots in the 2D-PAGE were weakly stained (Figure 1A). The spots excised from this region in the 2D-PAGE were identified as major egg antigen, annexin and troponin T proteins. Two spots corresponding to major egg antigen were identified in all the triplicate assays of both AW protein extracts (spots 14 and 15 in Figure 4 and Table 3). One extra spot of the major egg antigen was identified in all triplicate assays of AW-TEG protein extract (spot 42 in Figure 4 and Table 3). The presence of major egg antigen was confirmed in all of the IPG strip pH ranges using the INF serum pool (Table 2). Annexin was identified in all triplicate assays of the AW-TEG protein extract, but major egg antigen was co-extracted in the same spot as they co-migrate (spot 41 in Figure 4 and Table 3). Two spots corresponding to troponin T were identified in the AW-TOT protein extract (spots 31 and 32 in Figure 4 and Table 3). However, they were not present in all 2D-WB triplicate experiments, two experiments showed the spot 32 and one, the spot 31 (Figure 4 and Table 3). Other two spots, also immunoreactive only with the INF serum pool, were localized above 60 kDa, within a pH range 6.0 and 7.0 in the triplicate of AW-TEG protein extract, as pointed by arrows in the Figure 3. The corresponding proteins were identified as disulphide isomerase ER-60 precursor and filamin (spots 44 and 45, respectively, in Figure 4 and Table 3). From spot 21 indicated by the arrowhead in the Figure 3, two proteins were co-excised: actin and reticulocalbin (Figure 4 and Table 3), with low pI and at approximately 45 kDa. This spot was immunoreactive in the triplicate of AW-TEG protein extract and in one of the triplicate 2D-WB experiment of the AW-TOT protein extract when probed with INF serum pool (Figure 3). The transketolase (spot 24 in Figure 4 and Table 3) was identified as an immunoreactive spot exclusively when probed with INF serum pool in one 2D-WB experiment of the AW-TOT triplicate. However, in two 2D-WB experiments of the AW-TEG triplicate this protein was immunoreactive to both INF and NE serum pools.

**Table 3:** Immunoreactive spots to the pooled sera of *Schistosoma mansoni* infected, non-infected from endemic area and non-infected from non-endemic area volunteers identified in adult worm total and tegumental protein extracts.

SPOT number	PROTEIN DESCRIPTION	AW-TOT			AW-TEG		
		INF	NE	NI	INF	NE	NI
1	Fatty acid-binding protein (Sm14)	1	1	1	1	1	1
2	Superoxide dismutase [Mn]	1	1	1	1	1	1
3	14.3.3 zeta isoform	2	2	1	2	2	1
4	Glutathione S-transferase 28 kDa/Phosphoglycerate mutase	1	0	0	3	3	3
5	Glutathione S-transferase 28 kDa	3	3	2	3	3	3
6	Glutathione S-transferase 28 kDa	3	3	2	3	3	3
7	Phosphoglycerate mutase	-	-	-	3	3	3
8	Triose phosphate isomerase	2	2	1	3	3	3
9	Phosphoglycerate mutase	1	1	1	3	3	3
10	14.3.3 epsilon isoform	2	2	1	2	2	1
11	Hemoglobinase precursor	3	3	2	2	2	1
12	Malate dehydrogenase	1	1	0	3	3	1
13	Glyceraldehyde-3-phosphate dehydrogenase	1	1	1	3	3	3
**14**	**Major egg antigen**	**3**	**0**	**0**	**3**	**0**	**0**
**15**	**Major egg antigen**	**3**	**0**	**0**	**3**	**0**	**0**
16	Glyceraldehyde-3-phosphate dehydrogenase	2	2	2	3	3	3
17	Glyceraldehyde-3-phosphate dehydrogenase	3	3	3	3	3	3
18	Glyceraldehyde-3-phosphate dehydrogenase	3	3	3	3	3	3
19	Fructose 1,6-biphosphate aldolase/Phosphoglycerate kinase	3	3	2	3	3	3
20	Fructose 1,6-biphosphate aldolase	3	3	3	3	3	3
**21**	**Reticulocalbin/Actin-1**	**1**	**0**	**0**	**3**	**0**	**0**
22	Enolase (Phosphopyruvate hydratase)	3	3	2	3	3	3
23	Aldehyde dehydrogenase	2	2	1	3	3	3
24	Transketolase	1	0	0	2	2	0
25	Glutathione S-transferase 26 kDa	2	2	1	-	-	-
26	Troponin I (*S. japonicum*)	3	3	2	-	-	-
27	Myosin regulatory light chain	2	2	1	-	-	-
28	*Schistosoma mansoni*, expressed protein	2	2	1	-	-	-
29	Glyceraldehyde-3-phosphate dehydrogenase	1	1	1	-	-	-
30	Glyceraldehyde-3-phosphate dehydrogenase	1	0	0	-	-	-
**31**	**Troponin T**	**1**	**0**	**0**	-	-	-
**32**	**Troponin T**	**2**	**0**	**0**	-	-	-
33	Fructose 1,6-biphosphate aldolase	3	2	2	3	3	3
34	ATP synthase alpha subunit mitochondrial	3	2	2	-	-	-
35	Heat shock protein 70 (HSP70)	2	0	1	-	-	-
36	Glutathione S-transferase 28 kDa/Phosphoglycerate mutase	-	-	-	3	2	1
37	Hydroxyacylglutathione hydrolase (Glx II)	-	-	-	3	2	1
38	Four and A half lim domains	-	-	-	3	3	3
39	Short chain dehydrogenase/Four and A half lim domain	-	-	-	1	1	1
40	Eukaryotic translation elongation factor	-	-	-	0	2	0
**41**	**Annexin/Major egg antigen**	-	-	-	**3**	**0**	**0**
**42**	**Major egg antigen**	-	-	-	**3**	**0**	**0**
43	Aldehyde dehydrogenase	-	-	-	3	3	3
**44**	**Protein disulfide-isomerase ER-60 precursor**	-	-	-	**3**	**0**	**0**
**45**	**Filamin**	-	-	-	**3**	**0**	**0**
46	No hit	3	2	1	**2**	**2**	**1**

AW-TOT: adult worm total protein extract, AW-TEG: adult worm tegumental protein extract. INF: 2D-WB using pooled serum of *S. mansoni* infected individuals, NE: 2D-WB using pooled serum of non-infected individuals from endemic area and NI: 2D-WB using pooled serum of non-infected individuals from non-endemic area. (-): spots not immunoreactive in the corresponding protein extracts. The numbers (0, 1, 2 and 3) indicate how many experiments each spot was detected in the triplicate of the corresponding 2D-WB. In bold: spots immunoreactive exclusively in 2D-WB using INF serum pool. Highlighted line: spot immunoreactive exclusively to NE serum pool. Spot numbers are related to the Figure 4.

Only one immunogenic spot reacted exclusively with NE serum pool in two 2D-WB experiments of the AW-TEG triplicate. This spot of approximately 33 kDa and pI 8.0, as indicated by a dotted circle in Figure 3, corresponds to eukaryotic translation elongation factor (spot 40 in Figure 4 and Table 3). Although highly immunoreactive, this protein showed a low expression levels in AW-TEG protein extract (Figures 3 and 4).

**Figure 5:**
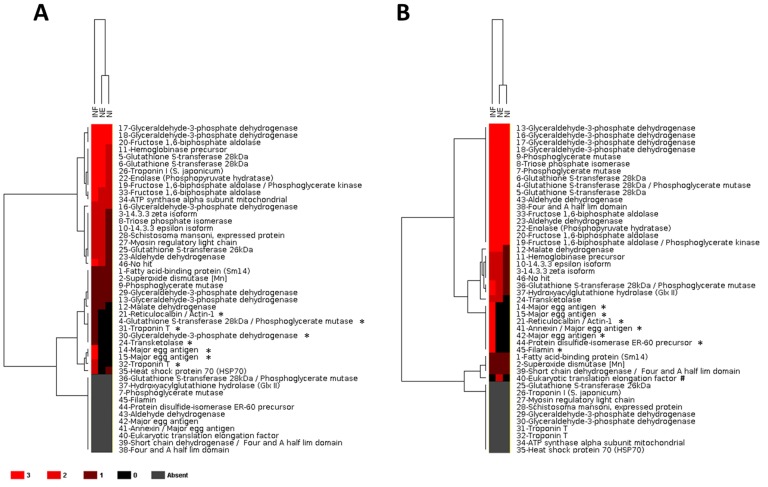
Heat map profile of immunoreactive *Schistosoma mansoni* adult worm proteins to specific pooled sera. S. *mansoni* adult worm total (A) and tegumental (B) protein extracts were analyzed by 2D-WB and the proteins identified by mass spectrometry were clustered according to the reactivity pattern against to *S. mansoni* infected (INF), non-infected from endemic area (NE) and non-infected from non-endemic area (NI) serum pools, in triplicate assays. Those proteins which reacted to the sera in all three assays were assigned by red color, whereas those with no detected reactivity, by black color. Proteins which were not identified in one of the protein extracts were shown by gray color. The immunogenic proteins which reacted exclusively to the INF serum pool are indicated by (*) and protein which reacted exclusively to the NE serum pool, by (#).

All the immunoreactive proteins were clustered by Euclidean distance with complete-linkage method according to reactivity pattern against INF, NE and NI serum pools in triplicate assays with AW-TOT (Figure 5A) and AW-TEG (Figure 5B). Using the heat map representation, the proteins which reacted exclusively with antibodies from the INF serum pool were clustered, and it was highlighted that the protein recognition pattern by NE and NI serum pools is closer than the NE and INF serum pools (Figures 5A and 5B).

### *In vitro* expression and western blotting analysis of recombinant *Schistosoma mansoni* major egg antigen and hemoglobinase

Two proteins identified in the 2D-WB experiments were selected for further validation of the immunoreactive pattern to the serum pools as recombinant protein expressed in a cell free *in vitro* system. The major egg antigen (MjE) was selected since it was identified in several spots immunoreactive only to the INF serum pool in the 2D-WB experiments (spots 14, 15, 41 and 42 in Table 3) using AW-TOT and AW-TEG protein extracts. The hemoglobinase precursor (Hem) was also selected since it was identified using all serum pools in the 2D-WB (spot 11 in Table 3).

**Figure 6:**
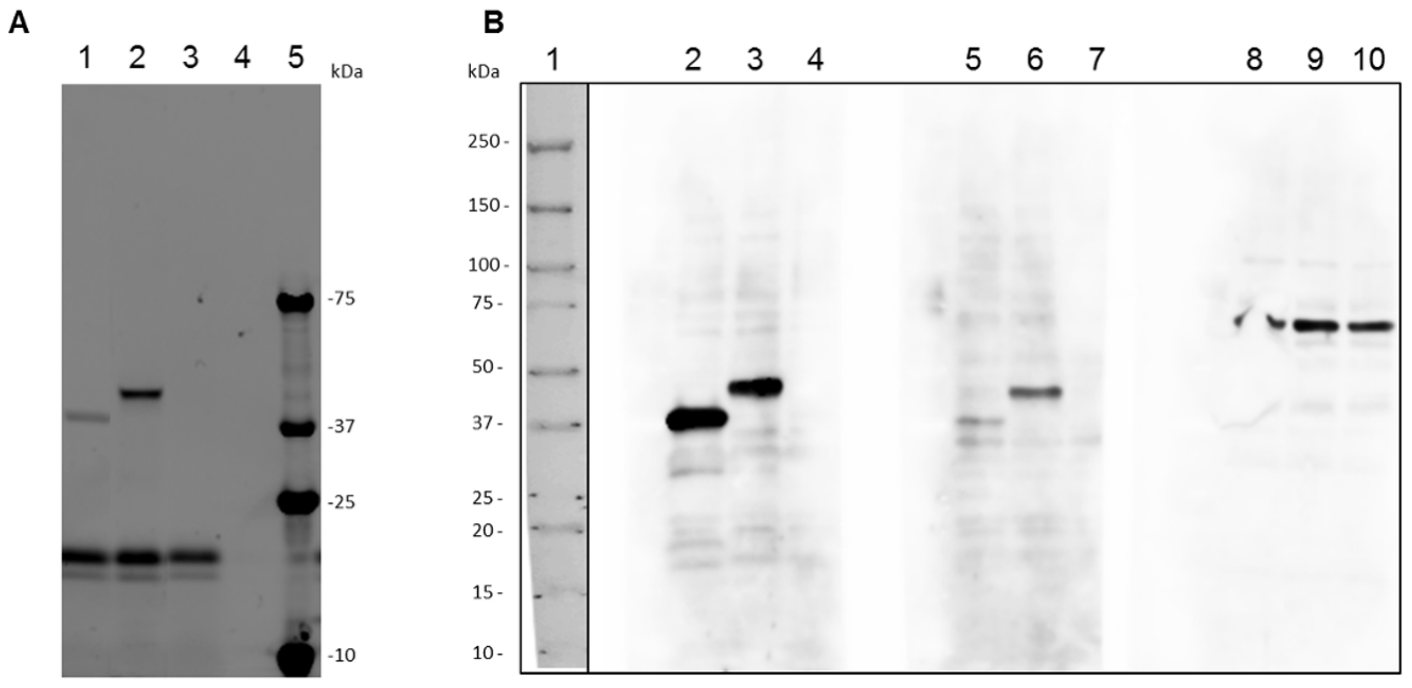
*In vitro* expression and western blotting analysis of recombinant *Schistosoma mansoni* major egg antigen and hemoglobinase proteins. A) *S. mansoni* major egg antigen (MjE) and hemoglobinase precursor (Hem) proteins, lanes 1 and 2 respectively, were expressed using TNT SP6 High-Yield Wheat Germ Protein Expression System and the FluoroTect Green_Lys_ labeled proteins were analyzed in SDS-PAGE 4–20%. The gel image was obtained with a laser-based fluorescent gel scanner. The negative control using no DNA template was indicated in lane 3 and using no DNA template and no FluoroTect Green_Lys_, in lane 4. Lane 5 contains the Precision Plus Protein Kaleidoscope molecular weight marker (BioRad). B) Western blotting analysis of the *in vitro* recombinant proteins probed with INF (2, 3, 4), NE (5, 6, 7) and NI (8, 9, 10) serum pools. The TNT wheat germ extract using MjE flexi-vector as DNA template was loaded in lanes 2, 5 and 8, and using Hem flexi-vector in lanes 3, 6 and 9. TNT wheat germ extract with no DNA template was loaded in lanes 4, 7 and 10. Rabbit anti-human IgG and anti-rabbit IgG HRP conjugated were used as secondary and tertiary antibodies. The membranes were developed using ECL Plus-Western Blotting Detection System and the proteins were visualized by chemiluminescence detection using a Fujifilm LAS-4000 Imaging System. Pre-stained Precision Plus Protein Kaleidoscope molecular weight marker was loaded in lane 1.

The coding region of the genes encoding these proteins was successfully amplified by PCR using *S. mansoni* adult worm cDNA library as template. The amplified fragments were inserted into pF3A WG (BYDV) Flexi Vector and the sequences confirmed by DNA sequencing. The *in vitro* coupled transcription-translation using the wheat germ system showed to be a suitable approach for expression of these schistosome proteins. The expressed proteins were visualized in the SDS-PAGE with the expected theoretical molecular mass, 40.3 kDa for MjE and 47.7 kDa for Hem, including the 6×His-tag (Figure 6A, lanes 1 and 2, respectively). In the negative control reaction, without DNA template, two protein bands of approximately 17 kDa were observed, as well as in the reactions using the plasmids containing the coding region of MjE and Hem. These bands correspond to newly synthesized biotinylated translation products of the TNT SP6 High-Yield Master Mix, which incorporate the labeled amino acid FluoroTect Green_Lys_ and are seen as background (Figure 6A, lane 3). In the reaction using no DNA plasmid template and no FluoroTect Green_Lys_ no fluorescent translation products were observed (Figure 6A, lane 4).

Western blotting analysis of recombinant proteins expressed by *in vitro* wheat germ expression system was performed using serum pool of *S. mansoni* infected (INF) and non-infected (NE) individuals from endemic area and also of non-infected volunteers (NI). The recombinant proteins maintained the recognition pattern of INF and NE serum pools used in the 2D-WB experiments, confirming their correct identification and the maintenance of the antigenic epitopes in the *in vitro* expressed proteins. MjE remained strongly reactive when tested against the INF serum pool, and was non- or weakly reactive with NE serum pool. The Hem recombinant protein reacted with both INF and NE serum pools, however, with greater reaction intensity against the INF serum pool. Neither MjE nor Hem was recognized by antibodies present in NI serum pool. Interestingly, the NI serum pool from UK volunteers reacted with a higher molecular weight protein from wheat germ extract, even in the negative reaction control using no DNA plasmid template. This reactive band was not observed using serum pools of individuals from schistosomiasis endemic area (Figure 6B).

## Discussion

*S. mansoni* adult worms can survive for decades in the hepatic portal system of the vertebrate host in spite of the parasites being constantly exposed to the host immune system and must, therefore, display effective strategies to evade the host immune response [69]. On the other hand, it has been well demonstrated that the development of protective immunity against schistosomes depends on both humoral and cell-mediated immunity [70]. In this study, we aimed to identify parasite antigens applying an approach that would enable the screening of a large number of antibody targets using serum of individuals residing in a schistosomiasis endemic area. This approach allowed the identification of antigens associated to the disease infection or resistance status.

A number of *Schistosoma* serological-proteomic studies have already been performed. However, this is the first which was conducted with *S. mansoni* using human sera, including sera of resistant and susceptible infection individuals living in an endemic area, allowing a more rational screening for new tangible human biomarker discovery. Although these studies have searched for schistosome vaccine candidates, most of them were based on animal models, with the caveat that they may not be directly translatable to humans [26]. In addition, similar studies with *S. haematobium* may not correlate with *S. mansoni*, as there is considerable variability in immune responses to crude antigens from both parasites [71], [72].

In this study we were able to identify 47 different antigenic proteins, a slightly larger number when compared to previous *S. mansoni* serological-proteomic studies. This can be attributed to the use of different IPG strip pH ranges and to the fact that all experiments in this study were performed in triplicate, increasing our chances of identifying new antigens. Although previous serological-proteomic studies were performed using protein extracts from other *Schistosoma* species and serum sources, some proteins were commonly identified such as HSP-70, enolase, GAPDH, triose phosphate isomerase, fructose-bisphosphate aldolase, glutathione S-transferase 28 kDa, 14-3-3 protein [30], [59], [73].

Among the immunoreactive proteins several are related to housekeeping metabolic pathways, such as glycolysis, gluconeogenesis and citric acid cycle; protein synthesis and proteolysis; transport; response to stress; detoxification process; cytoskeleton organization. In addition, some proteins that have already been tested as vaccine candidates, such as triose phosphate isomerase [74], glutathione S-transferase 28 kDa [75], fatty acid-binding protein (Sm14) [13] and superoxide dismutase [76] were also identified in this study.

For this work we decided to detect total immunoglobulins from sera of the different groups of individuals, since there is no clear mechanism of immunity in human schistosomiasis that defines a class of immunoglobulin as key for an effective immune response. Although some studies indicate IgE as an important immunoglobulin in schistosome post-treatment resistance, a vaccine trial for other helminth with an antigen that induces high IgE response showed significant adverse events [17], [77]. Therefore, we performed an analysis of all immunoreactive proteins regardless of immunoglobulin isotype using polyvalent anti-human Ig. Mutapi and co-workers [60] showed that there are qualitative and quantitative differences in *S. haematobium* antigen recognition profiles by human antibody isotypes (IgA, IgE, IgG1 and IgG4) although the majority of the adult worm antigens were recognized by all of these four isotypes. In earlier experiments, Delgado and McLaren [78] showed that IgG1 and IgG3 were involved in protective immunity against *S. mansoni* infection in mice. To address this possibility, we also preliminarily assayed for reactivity using anti-IgG1 and IgG3 in our serological screening. However we could not observe any significant differences in the antigenic pattern of INF, NE and NI sera recognition (data not shown).

Tegumental protein extract of *S. mansoni* adult worm was used to enrich our analysis with proteins that are directly exposed to host immune response. Although a large number of proteins were identified in both AW-TOT and AW-TEG protein extracts, there are still some differences between these extracts to be explored. Using 1D SDS-PAGE and LC-MS/MS, van Balkom and co-workers [40] identified 429 proteins, from which only 43 were specific to the tegument. All the tegumental specific proteins identified in our study were also identified by these investigators, and the proteins filamin and hydroxyacylglutathione hydrolase were exclusively identified in the tegumental protein fraction in both studies. Although we have used detergent buffer to solubilize integral membrane proteins for 2-DE in the AW-TEG, we were not able to identify immunoreactive proteins with transmembrane domains, as indicated in the SchistoDB database [67]. Despite extensive research, the large-scale analysis of membrane proteins by 2-DE remains a difficult task [79]. It is critical that other methods for membrane protein extraction that allow separation by 2-DE are developed.

Serum of non-infected volunteers from non-endemic schistosomiasis area showed immunoreactivity to some protein spots of AW-TOT and AW-TEG. It has been previously shown by Losada and co-workers [80] that the sharing of molecules among organisms is an expected finding since there are several functional molecules that are conserved during the process of evolution. These molecules may elicit immune responses between different species of various genera and is responsible for antigenic cross-reactivity. According to these findings, *Escherichia coli* and *Saccharomyces* antigens induce cross-reactivity with *S. mansoni* crude antigens, sharing T- and B-lymphocyte epitopes [81], [82]. Nevertheless, in our study 12 schistosome specific protein spots were detected only when sera of INF and NE were used.

Although most immunoreactive spots were visible in its corresponding 2D-PAGE, some highly immunoreactive protein spots were barely visible in the 2D-PAGE. Our analysis was conducted using only adult worm protein extracts, and there is a different level of protein expression during the life cycle of the parasite [31], [83]. Major egg antigen protein spots showed to be highly immunoreactive in the 2D-WB to the INF serum pool, although poorly expressed in AW-TOT and AW-TEG protein extracts. When a comparative analysis of the *S. mansoni* proteome among the life cycle stages was described by Curwen and co-workers [31], the major egg antigen was among the top 40 proteins expressed in egg protein extract (SEA). Therefore, the high level of major egg antigen immunoreactivity with the INF serum is probably due to the host immune response to this highly expressed protein in the parasite eggs that is also present in other life cycle stages.

As described by Wilson and Coulson [69] a single “magic bullet” has been shown not to be an efficient target for the development of a schistosomiasis vaccine. An antigen cocktail is suggested as a way to acquire protection. In line with this principle, in the current study we were able to cluster proteins with similar immunoreactivity pattern when using serum pools of infected or non-infected individuals. We observed that the major egg antigen, annexin, troponin T, filamin, disulphide-isomerase ER-60 precursor, actin and reticulocalbin proteins reacted exclusively with serum antibodies of the infected individuals and the eukaryotic translation elongation factor with antibodies present in the serum of the non-infected individuals from endemic area.

The major egg antigen, or Smp40, has been described as highly immunogenic in humans [84]. The cytokine profile obtained by PBMC from *S. mansoni* infected patients stimulated with purified Smp40 was associated with a reduction of granuloma formation and anti-pathology vaccine [85]. This protein was also previously suggested as a potential antigen to be used in an immunodiagnostic test, since it was effectively immunoprecipitated by *S. mansoni* infected human and chronically infected mouse serum [86], [87]. As for the Smp40 protein expression across the parasite life cycle, Nene and co-workers [86] showed that Smp40 could be easily detected by Western blotting assays in adults, cercariae, schistosomulum and egg stages, when probed with serum raised against a p40 fusion protein. Furthermore, van Balkom and co-workers [40] in their *S. mansoni* tegumental proteomics study have identified the Smp40 in both, tegumental and stripped worms protein fractions.

Filamin proteins are mechanical linkers for actin filaments and are also involved in signal transduction and transcription [88], [89]. Filamin was previously identified in an immunoscreening of cercarial cDNA library using IgG fraction of rabbit antiserum raised against immature female worms. A polyclonal antiserum specific to recombinant *S. mansoni* filamin revealed a tegument associated fluorescence in adult worms that reacted mainly with a band of 84 kDa, instead of the 280 kDa as indicated by the RNA sequence [90]. The filamin protein spot that was identified in this study using *S. mansoni* adult worm tegument protein extract was approximately 80 kDa, suggesting that they are the same protein. Previous studies using IgG specific antibodies to the recombinant filamin showed 36.6% killing of schistosomula *in vitro* and in a DNA vaccine immunization filamin induced a mean of 50% protection in mouse following challenge with adult worms by surgical transfer [90], [91].

Troponin T is one of the three protein subunits of the troponin complex that mediate Ca^2+^-regulation that governs the actin-activated myosin motor function in striated muscle contraction [92]. Troponin T was initially suggested to be a good candidate for use in the diagnostic test for *Taenia solium*. However, ELISA tests using pooled sera from cysticercosis-positive and negative patients showed disappointing results [93]. Troponin T was identified by van Balkom and co-workers [40] in a non-tegumental fraction of *S. mansoni* adult worm protein extract. Our study is in agreement with their study since we also identified it only in total protein extract, but not in tegumental protein extract. Another muscle protein identified in our study was actin. Actin was also previously observed to be reactive to prepatent infected mouse serum [94].

Reticulocalbin is a Ca^2+^-binding protein. It is localized in the endoplasmic reticulum, being involved in the secretory pathway, although its detailed role remains unknown. Overexpression of reticulocalbin may also play a role in tumorigenesis, tumor invasion, and drug resistance [95]. This protein has not been previously indicated as a diagnostic test or vaccine candidate for schistosomiasis.

Immunolocalization experiments and proteomic analysis of tegumental membrane preparations confirmed that annexin is a protein localized mainly in the tegument of *S. mansoni* schistosomula and adult worms [36], [96]. In this study, annexin was again identified exclusively in tegumental protein extract. *Schistosoma bovis* recombinant annexin was shown to be biologically active *in vitro*, with fibrinolytic and anticoagulant properties [97].

Significant attention is being given to the excretory system of schistosomes, since accumulating evidence suggests that it plays an important role in the host-parasite interaction. The *S. mansoni* cysteine protease ER-60 is one of four members of the parasite disulfide-isomerase protein family. It is expressed in adult worms and larvae excretory organs, suggesting a role for ER-60 during the host-parasite interaction [98], [99]. In this study, a disulphide-isomerase ER-60 precursor was recognized by INF serum in tegumental protein extract.

The *S. mansoni* eukaryotic translation elongation factor was the only protein identified in this study that reacted exclusively with antibodies present in NE serum pool. In addition to its canonical function in polypeptide chain elongation, the isoform eEF1A has been associated to viral propagation, apoptosis in metazoans, cytoskeleton organization and unfolded protein degradation [100]. Moreover, elongation factor 1b/d (31 kDa) has been shown to be immunoreactive to the sera of *Echinococcus granulosus* infected patients [101]. In our study, the immunoreactive spot corresponding to *S. mansoni* eukaryotic translation elongation factor was also approximately 31 kDa.

Although we have identified only one protein that was recognized exclusively by serum of the natural resistant individuals, which represents our major candidate for a vaccine development, all other 46 proteins identified also are candidates, once they were recognized by antibodies present in serum of infected individuals. The next step would be to assess the level of protection induced by these proteins in animal models.

Expression of recombinant proteins was important to confirm the antibody reactivity pattern, mainly for the proteins that were weakly stained in the 2D-PAGE, as it was performed with the major egg antigen. *In vitro* expressed *S. mansoni* major egg antigen was strongly recognized by the INF serum pool, maintaining a similar serum recognition pattern of the native protein which indicates the correct spot excision. Anti-human IgG were used in this Western blotting experiment, which means that major egg antigen is recognized by one of the IgG subclasses. However, in 2D-WB using anti-human IgG1 and IgG3, the spots corresponding to major egg antigen were no longer detectable using INF serum pool (data not shown). Additional studies with post-treatment sera and from individuals low stool egg counts will be important to test its potential, as well as of other promising candidates, as antigens for monitoring the cure and schistosomiasis infection. This will be particularly important for those individuals living in endemic areas for the detection of low infection rates. The antigens identified in this study may also be used as an immunodiagnostic test based, for example, on a qualitative predictive model able to distinguish the clinical status of the schistosomiasis endemic area residents.

*S. mansoni* hemoglobinase was also *in vitro* expressed in wheat germ cell-free system to confirm the serum recognition pattern of the 2D-WB experiment. While hemoglobinase was found to be immunoreactive to all the pooled sera used in 2D-WB, the recombinant protein showed to be associated with the INF and NE pooled sera, but not with the NI. It may be due to the use of anti-human IgG in the Western blotting instead of anti-human Ig's in the 2D-WB experiments, suggesting a specific reaction of other immunoglobulin class in 2D-WB experiments.

Serological-proteomics is a demanding methodology with a number of arduous steps, consequently the reproducibility in 2D-WB experiments is a difficult task [102], causing missed immunoreactive spots in the triplicate experiments and among the immunoblots. Furthermore, matching the immunoreactive spots of 2D-WB to stained spots in 2D-PAGE gels used to excise the spots to MS/MS analyses is not a trivial task. To precisely locate immunoreactive spots in the 2D-PAGE gels, the molecular weight, pI and the distribution pattern of spots neighboring the spot of interest had to be taken into account. The same difficulty occurs when matching the spots among the immunoblots. It must also be taken into account that spots do not represent proteins but rather protein species with post-translational modifications, partly degraded polypeptides, or may be splicing variants or paralogues genes. Additionally, spots often contain several different proteins or protein species [102].

Despite these difficulties, serological-proteomics seems to be a good approach to characterizing the host immune response profile to parasite antigens in a large-scale analysis, overcoming the case-by-case and empirical science used in the past and providing prominent antigens for the development of new schistosomiasis diagnostics and vaccines. The manageable repertoire of *S. mansoni* antigens identified in this study warrants further investigation by profiling the antibody response of a larger panel of individual sera using different immunoglobulin classes/subclasses. Understanding the human immune response associated with the infection/protection profile to these antigens represents a huge step towards the improvement of diagnostic tools and development of vaccine against schistosomiasis, using not only one but multiple antigens.
